# An individual data-driven virtual resection model based on epileptic network dynamics in children with intractable epilepsy: a magnetoencephalography interictal activity application

**DOI:** 10.1093/braincomms/fcad168

**Published:** 2023-05-25

**Authors:** Pablo Cuesta, Ricardo Bruña, Ekta Shah, Christopher Laohathai, Stephanie Garcia-Tarodo, Michael Funke, Gretchen Von Allmen, Fernando Maestú

**Affiliations:** Department of Radiology, Rehabilitation and Physiotherapy, Universidad Complutense de Madrid, Madrid, 28040, Spain; Center for Cognitive and Computational Neuroscience, Complutense University of Madrid, Madrid, 28040, Spain; Instituto de Investigación Sanitaria San Carlos (IdISSC), Madrid, 28040, Spain; Department of Radiology, Rehabilitation and Physiotherapy, Universidad Complutense de Madrid, Madrid, 28040, Spain; Instituto de Investigación Sanitaria San Carlos (IdISSC), Madrid, 28040, Spain; Department of Pediatrics, McGovern Medical School, The University of Texas Health Science Center at Houston, Houston, TX 77030, USA; Department of Neurology, Saint Louis University, Saint Louis, MO 63110, USA; Département de la femme, de l'enfant et de l'adolescent, Hôpital des Enfants - Hôpitaux Universitaires de Genève, Geneva, 1211 Genève 14, Switzerland; Department of Pediatrics, McGovern Medical School, The University of Texas Health Science Center at Houston, Houston, TX 77030, USA; Department of Pediatrics, McGovern Medical School, The University of Texas Health Science Center at Houston, Houston, TX 77030, USA; Center for Cognitive and Computational Neuroscience, Complutense University of Madrid, Madrid, 28040, Spain; Instituto de Investigación Sanitaria San Carlos (IdISSC), Madrid, 28040, Spain; Department of Pediatrics, McGovern Medical School, The University of Texas Health Science Center at Houston, Houston, TX 77030, USA; Department of Experimental Psychology, Cognitive Processes and Speech Therapy, Universidad Complutense de Madrid, Madrid, 28040, Spain

**Keywords:** magnetoencephalography, interictal activity, computational neurosurgery, children epilepsy

## Abstract

Epilepsy surgery continues to be a recommended treatment for intractable (medication-resistant) epilepsy; however, 30–70% of epilepsy surgery patients can continue to have seizures. Surgical failures are often associated with incomplete resection or inaccurate localization of the epileptogenic zone. This retrospective study aims to improve surgical outcome through in silico testing of surgical hypotheses through a personalized computational neurosurgery model created from individualized patient’s magnetoencephalography recording and MRI. The framework assesses the extent of the epileptic network and evaluates underlying spike dynamics, resulting in identification of one single brain volume as a candidate for resection. Dynamic-locked networks were utilized for virtual cortical resection. This in silico protocol was tested in a cohort of 24 paediatric patients with focal drug-resistant epilepsy who underwent epilepsy surgery. Of 24 patients who were included in the analysis, 79% (19 of 24) of the models agreed with the patient's clinical surgery outcome and 21% (5 of 24) were considered as model failures (accuracy 0.79, sensitivity 0.77, specificity 0.82). Patients with unsuccessful surgery outcome typically showed a model cluster outside of the resected cavity, while those with successful surgery showed the cluster model within the cavity. Two of the model failures showed the cluster in the vicinity of the resected tissue and either a functional disconnection or lack of precision of the magnetoencephalography–MRI overlapping could explain the results. Two other cases were seizure free for 1 year but developed late recurrence. This is the first study that provides in silico personalized protocol for epilepsy surgery planning using magnetoencephalography spike network analysis. This model could provide complementary information to the traditional pre-surgical assessment methods and increase the proportion of patients achieving seizure-free outcome from surgery.

## Teaser

Cuesta *et al*. present, in a retrospective study, an in silico personalized (no use of group data) protocol for epilepsy surgery planning using magnetoencephalography spike network analysis. The performance of the model in a cohort of 24 paediatric patients with focal drug-resistant epilepsy reached high accuracy, sensitivity and specificity scores.

## Graphical Abstract

**Graphical abstract:**
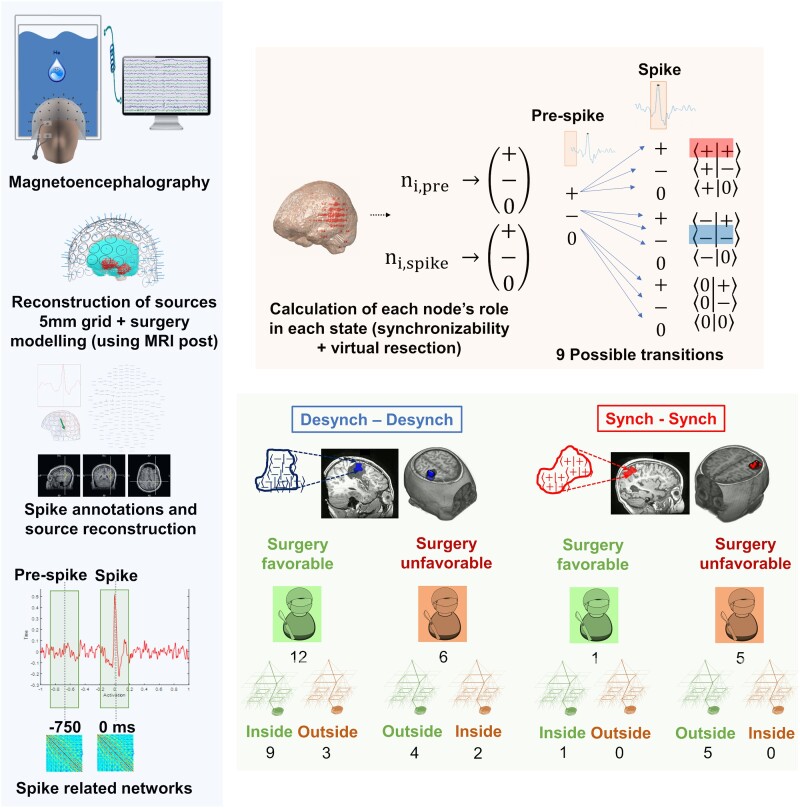

## Introduction

Epilepsy affects more than 50 million people worldwide.^1^ The impact of epilepsy ranges from impaired quality of life^2^ and reduced life expectancy.^3^ Medication resistance occur in 30–40% of patients,^4^ and surgical option continued to be recommended, especially in focal onset epilepsy.^5,6^ However, 30% to 70% of patients who underwent epilepsy surgery continued to have residual seizures, suggesting an incomplete resection or inaccurate localization of epileptogenic zone.^7,8^

Many advances in neuroimaging techniques and electrophysiological recordings have contributed to increasingly precise identification of the epileptogenic tissue and network.^9^ Invasive intracranial electroencephalographic recordings are often required to confirm localization hypothesis and elucidate discordant findings, creating an additional procedural step which increases operative risk and hospitalization cost. Moreover, invasive intracranial recording has limitations as the procedure explores limited brain regions and emphasizes the localization of seizure onset but does not provide complete information regarding broader epileptic networks. Many patients continued to have seizures with this approach. We view that parallel refinements of additional investigative procedures are needed to limit procedural risks and improve patient’s surgical outcome. To achieve this, we perceive that identification of surgical targets through conceptual understanding of epilepsy as a network can supplement the conventional methodologies that rely on identification of seizure onset.

Epileptic phenomena (ictal or inter-ictal) are transient alterations of brain dynamics, with marked local and distant changes in the relationship between network nodes that can be detected through both non-invasive [EEG; magnetoencephalography (MEG)] and invasive recordings.^10,11^ Several recent studies have used connectivity analyses to determine the extent of global and regional epileptic networks in both focal^12-14^ and generalized seizures.^15^ When accurately identified, the disruption of the epileptogenic network can be achieved through minimal resection of the primary or leader network nodes, with good seizure freedom and functional outcome.^16^ Studies have attempted to dissect the functional epileptic network and identify the major determinants of seizure generation and spread.^14,17-23^ This ‘network theory’ approach led to development of in silico models for testing and prediction of surgical outcome.^10,16,24-27^ We pay particular interest to the framework developed by Bassett and colleagues [– that is virtual cortical resection (VCR)]^28,29^ where graph metrics [– that is network synchronizability (S)] of seizure onset and surrounding tissue were used to model epileptic network and assess the role in epileptic triggering of each brain regions. The notion of distinct S between the seizure and pre-seizure states, and the division of the network into seizure generating and regulatory zones, highlights a spatially elaborate and temporally evolving pattern.^27,30^ This dynamic process challenges the conceptualization of a single epileptic network model and provides the identification of diverse elements central to seizure evolution and cessation.

Epileptic network organization is complex and demands data with high spatio-temporal resolution for an adequate assessment. Conventional scalp EEG data is limited by volume conductor and spatial resolution. Although stereoelectroencephalography (SEEG) data can provide information regarding involvement of deep brain structures, the extent of measurement is limited by electrode placements and sampling. MEG is a validated non-invasive procedure that provides assessment of cortical sources of epileptogenic activity, as well as deeper foci (or network components), with high spatial sampling and temporal resolution.^31-33^ With its broad spatial resolution and less propensity to be affected by tissue conductivity, MEG is an effective tool to assess the epileptic network dynamics. The accuracy of MEG in the detection and measurement of epileptic network has been validated against SEEG.^34^

We propose a novel approach of merging epileptic network model obtained from MEG data with computational models of epilepsy surgery (referred to as ‘computational neurosurgery’) to create for a framework of surgical planning in silico that allows for testing of different interventions/targets and their possible outcomes before the surgical procedure. Transitions-locked networks were used to identify targets associated with spike triggering to develop VCR framework.^28,29^ Furthermore, in contrast to the non-specific computational approach utilizing group data,^29^ we have developed a personalized computational neurosurgery protocol that would be tested individually against a cohort of paediatric patients who underwent epilepsy surgery. This is the first study to create such personalized in silico protocols for epilepsy surgery through non-invasive electrophysiological data.

## Materials and methods

### Patients’ dataset and inclusion criteria

All paediatric MEG studies performed at Children’s Memorial Hermann Hospital (Houston, TX, USA) between January 2013 and December 2017 were retrospectively reviewed. Patients who received outpatient MEG study with at least 30 epileptic MEG spikes, and subsequently underwent resective surgery at Children’s Memorial Hermann Hospital, were included. Exclusion criteria include patients with generalized MEG discharges (or rare or no interictal activity), existing vagus nerve stimulator at the time of MEG testing, received MEG as inpatient or had less than 2 years of post-surgical follow-up period.

A total of 24 paediatric patients who met the study criteria were identified. Relevant medical information, including outpatient documentations, hospitalization records, operative notes, non-invasive EEG data and neuroimaging studies, were reviewed. Seizures were classified according to the 2017 International League Against Epilepsy seizure type classification.^35^ Engel classification^36^ was used to categorize post-surgical outcome, where Engel 1 defines a favourable outcome.

### Clinical selection of epileptiform MEG activity

MEG recordings were performed in accordance with the American Clinical MEG Society clinical practice guidelines.^37^ All studies were conducted in the supine position inside a magnetically shielded room, using a 306-channel whole head MEG system (Triux, MEGIN, previously known as Elekta-Neuromag, Helsinki). Simultaneous EEG was recorded using a 32- or 64-channel electrode cap, depending on head size. Before recording, the positions of three external fiduciary points, five head position indicator coils, all EEG electrodes and several hundred head shape points were obtained.^38^ Continuous head position monitoring was performed to account for any shift in head position in all recordings. Acquisition parameters for MEG/EEG included a sampling rate of 1000 Hz and a band-pass filter of 0.1–330 Hz. Raw data was subsequently processed using a spatio-temporal signal space separation method^39-41^ that compensates for significant head movements and suppresses interferences from nearby magnetic sources. Data processing and analysis were conducted, using the vendor software packages (DANA and MaxFilter by MEGIN, Helsinki). The MEG and EEG data were visually inspected for focal slowing and epileptiform discharges by a magnetoencephalographer and an epileptologist. Individual epileptic MEG discharges were sourced modeled using multiple equivalent current dipole model in the patient’s coregistered brain MRI in accordance with American Clinical MEG Society practice guidelines and were retained for connectivity analysis if statistically significant (reduced Chi-square >1.0 and <2.0, confidence volume 1000 mm^3^, source strength 100–500 nAm, goodness of fit > 75%). Although our clinical MEG reporting standard includes the comparison of MEG and its EEG correlates: significant peak latencies, signal amplitudes and source configurations, in this study only MEG was employed in the network analysis to maximize the resolution of high frequency bands and to be able to use a simpler headmodel (1 layer) for the reconstruction of the time series at the sources level.

The sequential progress of the present piece of work went through two different phases. In the initial approach, we focused on developing a virtual surgery that agreed with real clinical cases. To do so, the first test design (henceforth called ‘Step 1’, see Table 1) included the anatomical information of the resection cavity obtained from post-surgical MRI in the construction of the spike-associated network (SAN). This way, we could easily check whether the possible solutions were located within the resected cavity. It is important to note that the model exposed in this study works patient by patient, without any group data or previous information. Once the structure of the model was defined, the second test design (henceforth called ‘Step 2’, see Table 2) was carried out as a blinded performance test of the virtual surgery model. The relevance of this difference is later addressed in the SAN definition. The patient’s outcome was blinded for the analysis group (Madrid team).

**Table 1:** Demographic information of all patients involved in Step 1

ID	Sex	Age of epilepsy diagnosis	Age at surgery	Surgery	Aetiology	Engel (2 years)	Outcome
1	F	2 years	17 years	L-Fr-Resc, L-Temp-Lob + L-AHyp	Hippocampal sclerosis	1a	Favourable
2	F	7 years	9 years	R-Temp-Par-Occ-Lob	Perinatal stroke	1a	Favourable
3	F	11 weeks	13 months	L-Temp-Occ-Resc	FCD	4	Unfavourable
4	F	11 weeks	18 months	L-Par-Temp-Occ-Resc	FCD	1a	Favourable
5	F	3.5 years	8 years	R-Inf-Ft-Par-Resc	Perinatal stroke	1a	Favourable
6	M	14 months	3 years	R-Occ-Resc	FCD, migrational anomaly, genetic	4	Unfavourable
7	M	14 months	3.5 years	R-Occ-Lob	FCD, migrational anomaly, genetic	1a	Favourable
8	F	11 years	14 years	L-Ant-Temp-Lob + L-AHyp + L-Occ-Top	FCD, hippocampal sclerosis, potentially genetic	1a	Favourable
9	F	6 years	16 years	R-Par-Resc	Migrational anomaly	1b	Favourable
10	F	10 months	3 years	L-Inf-Par-Temp-Occ-Lob	Perinatal stroke, migrational anomaly, genetic, hippocampal sclerosis	3	Unfavourable
11	M	10 years	17 years	L-Ant-Temp-Lob + L-AHyp	FCD, hippocampal sclerosis, genetic	1b	Favourable
12	M	9 days	10 months	L-Par-Tub	Tuberous Sclerosis Complex	1a	Favourable
13	F	9 years	10 years	L-Ant-Temp-Lob + L-Hyp	FCD, hippocampal sclerosis	2	Unfavourable
14	M	6 months	18 months	L-Temp-Occ-Resc	Tuberous Sclerosis Complex	1a	Favourable

Step 1 was used as an experimental design to test the model within a surgery already carried out. For the patients involved in Step 1, the network of interest included all nodes within the resected area, regardless of whether they were selected in the automatic network configuration.

L, left; R, right; Inf , inferior; Ant, anterior; Fr, frontal; Temp, temporal; Par, parietal; Occ, occipital; Ins, insular; AHyp, amygdalohippocampectomy; Hyp, hippocampectomy; Resc, resection; Lob, lobectomy; FCD, focal cortical dysplasia). Engel classification from.^36^.

**Table 2:** Demographic information of all patients involved in Step 2

ID	Sex	Age of epilepsy diagnosis	Age at surgery	Surgery	Etiology	Engel (2 years)	Outcome
15	F	6 years	10 years	L-Temp-Par-Resc	FCD	1a	Favourable
16	F	Infancy	6 years	L-Temp-Par-Occ-Resc	Tuberous Sclerosis Complex	4	Unfavourable
17	F	Infancy	7 years	L-Ft-Resc	Tuberous Sclerosis Complex	4	Unfavourable
18	F	6 years	8 years	L-Par-Resc	FCD, genetic	4	Unfavourable
19	F	6 years	10 years	L-Par-Ins-Resc	FCD, genetic	4	Unfavourable
20	M	8 months	3 years	L-Par-Occ-Resc	FCD, genetic	2	Unfavourable
21	M	13 years	20 years	R-Temp-Par-Resc	FCD	1a	Favourable
22	F	8 years	16 years	L-Par-Occ-Resc-Disc	Perinatal stroke	1a	Favourable
23	F	5 weeks	17 months	L-Ft-Temp-Resc	Tuberous Sclerosis Complex	4	Unfavourable
24	F	1 year	17 years	R-Fr-Resc	FCD, peri-versus antenatal stroke	4	Unfavourable

Step 2 was used as an experimental design that emulates a surgery planification. For these patients, the network of interest was not forced to include all nodes within the resected area (only those nodes automatically selected were included).

L, left; R, right; Inf, inferior; Ant, anterior; Fr, frontal; Temp, temporal; Par, parietal; Occ, occipital; Resc, resection; Disc, disconnection; FCD, focal cortical dysplasia. Engel classification from^36^.

### Pre-processing

The resting state signals were automatically scanned for movement and myogenic artefacts using FieldTrip software^42^ and visually confirmed by a MEG expert. Independent component analysis-based procedure was employed to remove the cardiogenic artefacts. The remaining artefact-free data were segmented in 2 s trials using the spike onset as the time reference (to be able to perform time-locking analysis). Accordingly, segments were defined as −1 to 1 s, where t = 0 corresponded with the spike onset. MEG time series were filtered into 12 frequency bands using 1500th order finite impulse response filter designed with Hamming window, and a two-pass filtering procedure, prior to source data calculation [theta (4–8 Hz); alpha (8–14 Hz); beta (15–30 Hz); gamma1 (30–45 Hz); gamma2 (45–55 Hz); gamma3 (65–75 Hz); gamma4 (75–85 Hz); gamma5 (85–95 Hz); gamma6 (95–105 Hz); gamma7 (105–115 Hz); gamma8 (125–135 Hz); gamma9 (135–145 Hz)]. Gamma band was subdivided into nine sub-bands mirroring the ones used on the papers of Khambhati *et al.*^28^ and Kini *et al*.,^29^ Data were filtered using 2 s of recording at each side as padding to avoid edge-related artefacts.

### Source reconstruction

The individual pre-surgery T1 image from each participant was segmented into white matter, grey matter and CSF using SPM12 and the Unified Segmentation algorithm.^43^ We defined the individual source model as a regular 3D grid with 5 mm of separation covering the whole head, and only those source positions laying in grey matter were considered (between 5000 and 6000 nodes, henceforth called sources). Forward model utilized a single-shell interface determined by the brain volume (union of white matter, grey matter and CSF), which was combined with the source model and the sensor definition to generate a lead field using a modified spherical solution.^44^ Source reconstruction was performed independently for each subject and frequency bands using a linearly constrained minimum variance beamformer.^45^ Beamforming filters were estimated jointly for magnetometers and gradiometers using normalized (by sensor type) lead fields, the average covariance of the corresponding trials and a regularization factor of 15% of the signal power.

### SAN definition

The analysis procedure started with the automatic construction of a SAN; a network defined for those sources (chosen among the starting ∼5500) whose activity seemed to be the most associated with the appearance of the spike. The SAN construction methodology segregated the patients of this study in two samples, each one corresponding with a specific clinical scenario: (i) in the Step 1, the information obtained from the postsurgical MRI was used to force the inclusion of the sources within the resection cavity in the SAN which would be corresponding with an already planned surgery; (ii) in the Step 2, the information of the postsurgical MRI was not considered. The rationale behind this decision was to mimic the scenario where the resection area is yet to be defined and no assumption exists. For the Step 1, the automatic algorithm for the SAN construction consisted of a five-step procedure: (i) the activity for each candidate source was reconstructed between 2 and 55 Hz using the corresponding beamformer filter. The band was defined to allow the visual inspection of the spike-related activity; (ii) the candidate sources were ranked, per each spike/trial, attending to the amplitude at the moment of the spike onset; (iii) only sources ranked within the first 1000 in Step 2 in at least 75% of the trials were kept; (iv) the SAN was defined by the union of the 5% of the sources with higher rank after Step 3, plus all the sources in the resection area (defined from the post-surgical MRI); and (v) a spatial clustering procedure (following the methodology used by Fieldtrip^42^) was applied to remove those sources that were not part of a cluster that had at least five nodes. The minimum size was set to agree with the one fixed for the candidate cluster of the results (see ‘The computational neurosurgery model’ section). In the case of Step 2, all steps were identical but the exception of Step 4, where the postsurgical MRI was not employed. Therefore, since only sources of the SAN can be used for the construction of the candidate cluster, for the Step 2 patients the possibility of getting clusters inside or partially inside the resection cavity decreased. This limitation in the extension of the SAN was assumed as a drawback of the blind experiment but required to represent a realistic clinical scenario where the resection would have not been determined. The resulting SANs in both samples contained around 500–600 sources.

### Functional connectivity computation

We assess the functional connectivity (FC) of the SAN in two different brain states (each one lasting 100 ms), defined as follows: (i) a pre-spike state centred at 750 ms before the spike onset and (ii) a spike-onset state centred at the spike onset. The FC was computed phase locking value (PLV) by means of a smoothed version of the Lachaux *et al.*^46^ methodology. The PLV in the original Lachaux procedure was obtained by concatenating equivalent time-locked data samples to form the data segments on which the averages of the phase-differences pairs were computed. In the case of this piece of work, the methodology employed a window of 100 ms length to ensure the consistency of the PLV scores corresponding to low frequency bands. Consequently, every PLV value was computed with a data segment whose length corresponded to number of spikes (trials)×100 ms. The use of PLV was based on three main reasons: (i) its high similarity to coherence, which was the FC method used by Khambhati *et al.*^28^ and Kini *et al.*^29^; (ii) the possibility to be computed very fast even with high dimensional data thanks to an algorithm developed by our group;^47^ and (iii) because its well demonstrated test-retest reliability.^48,49^ To get a better estimation of the FC over the temporal window, the instantaneous phases of the different spike-related epochs were concatenated, and PLV values were estimated along the whole 100 ms window. As a result, we obtained a set of two symmetrical PLV matrices (pre-spike and spike) per frequency band.

### Virtual resection methodology

**Figure 1:**
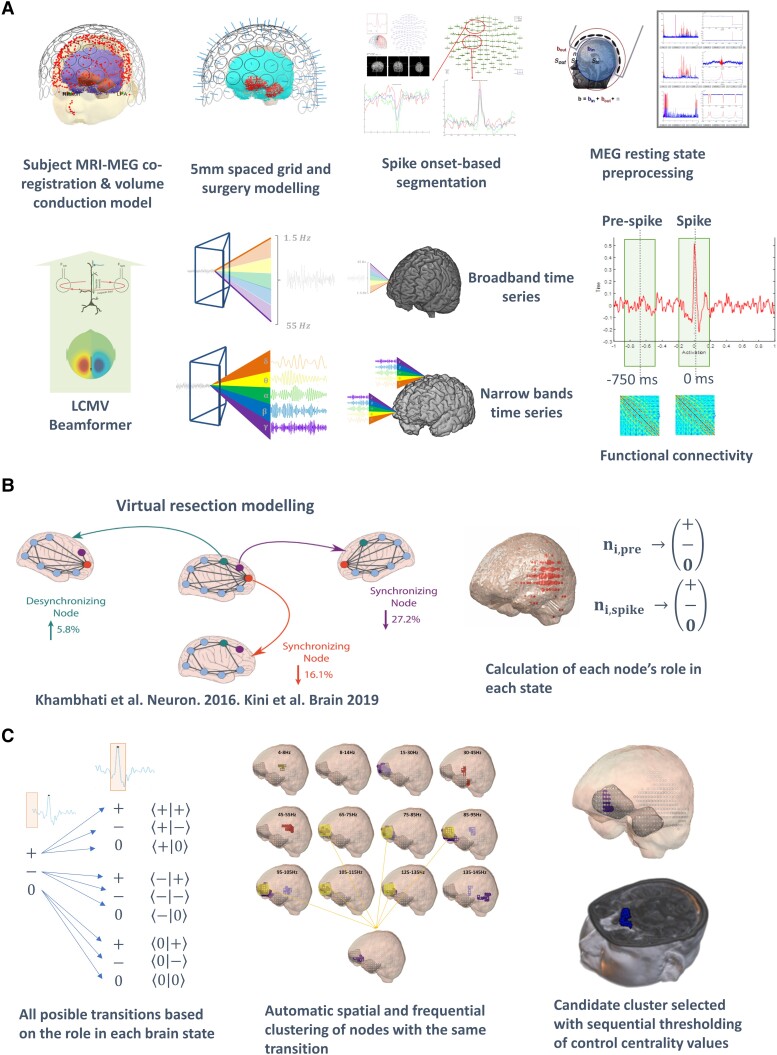
**Scheme representing the methodological pipeline.** (**A**) Processing of MEG signals, including the source reconstruction of clean spike-related data and calculation of the functional connectivity of two consecutive brain steps. (**B**) Virtual resection method. Extraction of the role of each source in the network dynamics for each state using a virtual resection procedure as published in.^28^ (**C**) Definition of brain regions with identical transition dynamic between both states across several frequency bands. Using a sequential thresholding procedure, the first cluster to emerge constitutes the candidate solution

The modelling of the network’s response to a potential resective surgery was assessed through a methodology termed ‘VCR’ (see Fig. 1B), designed by other groups and applied on similar studies to the one presented here.^28,29^ The VRC makes use of the metric S,^50^ a graph measure that accounts for the stability of the network by evaluating how easily the neural activity can synchronize through the entire network. The VCR procedure is started by the determination of the S in the network. Then, iteratively per each source, the algorithm performs a virtual deletion of the corresponding source and re-evaluates the S value of the network. The VCR was carried out source-wise for both brain states and the 12 frequency bands, characterizing the role in the network topology of each one of the sources, brain states and frequency bands. This characterization was weighted by means of the control centrality (CC),^28^ defined as: CCi = Si-S/S, where S and Si correspond with the S before and after the source removal. The CC represents the per-one change in S due to the deletion of the corresponding source. A positive CC value implies that the S increased after the source removal, pointing out, for the corresponding source, a desynchronizing role in the network. Conversely, if the S decreased after source removal, the source was categorized as synchronizing. Therefore, the VRC procedure ended up with a CC matrix whose dimensions were: number of sources × per brain state (2) and per frequency band (12).

### The computational neurosurgery model

To pinpoint those brain areas with a capital role on the diffusion of the epileptogenic activity, we created an evolution model (see Fig. 1C), between the pre-spike and spike-onset brain states. For each frequency band, we used the CC vectors obtained in the VCR to label the sources in the SAN in each state as synchronizing (+ in Fig. 1), desynchronizing (−), or bulk (0). Based on this triphasic model, the evolution methodology assessed all nine possible itineraries for a specific source of the SAN throughout the epileptic process in each frequency band. Bulk sources were defined as those whose CC values fell within the 10% (5%, two tails) closer to 0. Finally, we rank the candidate solutions by means of a sequential thresholding procedure. For each threshold (from 1% to 100% of the biggest CC values, in steps of 1%, using two tails), only those CC values with weights within the corresponding bigger % were kept in the CC vectors, whereas all the remaining CC values were discarded. Then, we used an automatic multidimensional clustering procedure (enforcing spatial and frequency adjacency) to extract those regions with a locked dynamic trajectory through the generation of the epileptiform activity. The clustering was carried out by means of the function *bwlabeln* of the Image Processing Toolbox of Matlab, following the method used by Fieldtrip and the cluster-based permutation test algorithm.^42,51^ The physical clustering condition was fixed to have a minimum size of 15 sources and require that the trajectory of the sources would be locked at least for 4 consecutive frequency bands (frequential clustering). Candidate cluster would be defined as a brain region (set of spatially adjacent nodes) whose electrophysiological activity behaves with an specific transition profile consistently along 4 consecutive frequency bands through the generation of the epileptiform activity (modelled as the evolution from the pre-spike to the spike-onset brain states). The sequential thresholding iteration consisted of successively adding more nodes (starting for those nodes with higher CC scores (in absolute value)) and testing for possible clusters till one arises. If after including all nodes, no cluster emerged, then the minimum size condition was lowered in five sources, and the sequential process started again.

### Statistical analysis

To evaluate the model performance, three measures or reliability were calculated using the available information (– that is localization inside/outside the resected cavity and the patient outcome after the epilepsy surgery): (i) accuracy: probability of correct predictions; (ii) specificity: likelihood of a negative result to be truly negative; and (iii) sensitivity: likelihood of a positive result to be truly positive. In the context of the present study, true positives (TPs) were defined as cluster inside + favourable outcome, false positives (FPs) were defined as cluster inside + outcome unfavourable, true negatives (TNs) were defined as cluster outside + outcome unfavourable and false negatives (FNs) were defined as cluster outside + favourable outcome. Consequently, sensitivity was computed as TP/(TP + FN); specificity as TN/(TN + FP); and accuracy as TP + TN/(TP + TN + FP + FN). Fisher’s exact test was computed to test the randomness of the results for each step sample and for the whole population of this study.

### Patients outcome

On one hand, a model success was characterized by (i) good postsurgical outcome and model solution with the surgical resection cavity or (ii) unfavourable postsurgical outcome and model solution outside the resection cavity. On the other hand, a model failure was characterized by (iii) good postsurgical outcome and model solution inside the surgical resection cavity or (iv) unfavourable postsurgical outcome and model solution outside the resection cavity.

## Results

Step 1, where the information of the resection cavity in the post-surgical MRI was forced in the conformation of the SAN, included 14 patients (see Table 1). Step 2, where post-surgical information was not employed, included 10 patients (see Table 2). The methodological pipeline (see Fig. 1), which involved the computation of FC in two consecutive brain states in 12 non-overlapping frequency bands, allowed for candidate cluster identification.

### Step 1

#### Calibrating model performance

**Figure 2:**
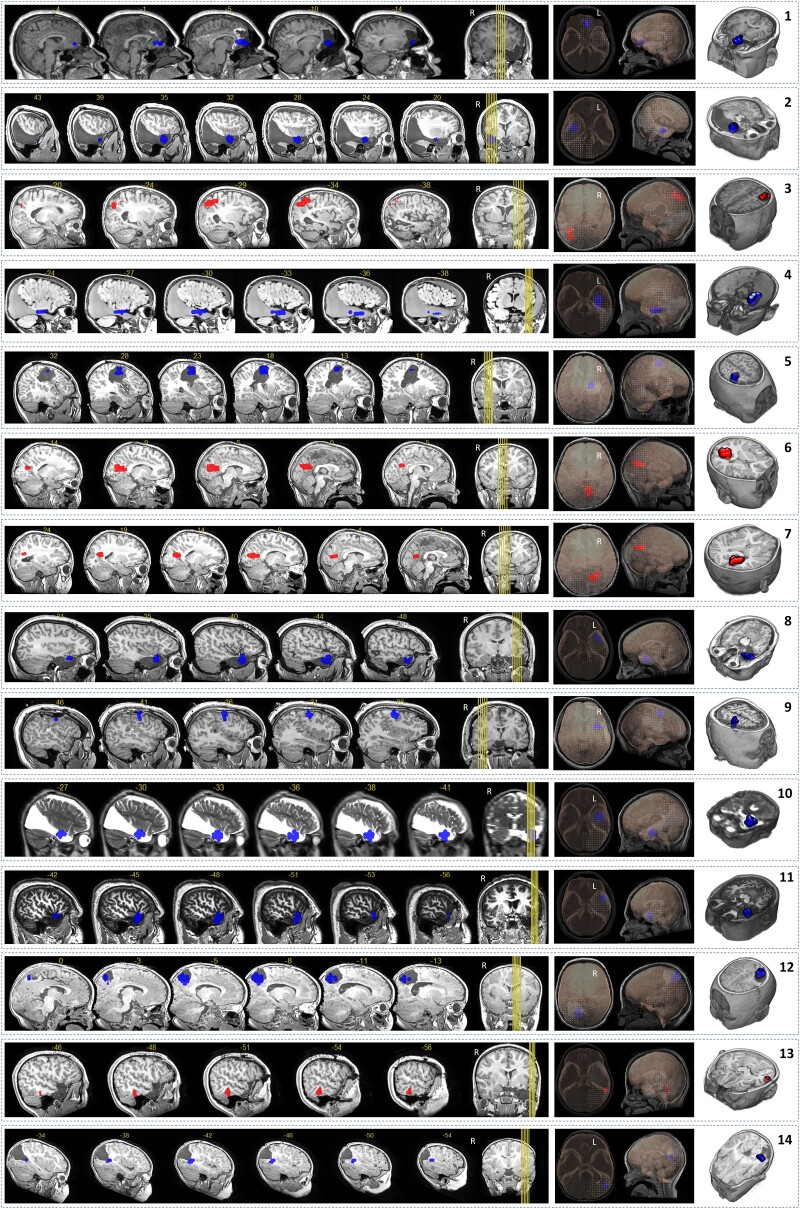
**Candidate clusters for the patients analysed in Step 1.** The figure displays in each row the result for each one of the 14 patients involved in the first experiment (Step 1). All different views show the same information, the cluster location. In the centre figures, the 3D model shows the headmodel of the corresponding patient. Clusters in the rows 1,2, 4, 5, 8, 9, 10, 11, 12 & 14 (highlighted in blue) indicate brain regions whose transition between the pre-spike period and the spike-onset period was defined by a desynchronizing–desynchronizing pattern. Clusters in the rows 3, 6, 7 & 13 (highlighted in red) represent brain regions with a synchronizing–synchronizing profile

**Figure 3:**
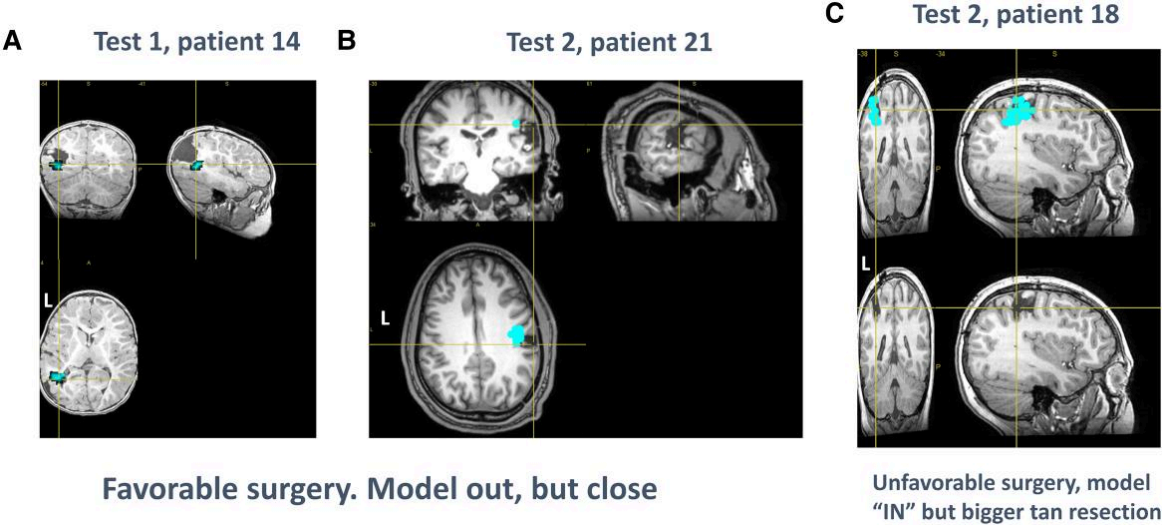
**Individual results of interest.** (**A** and **B**) Patients with favourable surgeries who showed a candidate cluster located outside the surgery cavity. (**C**) Patient with unfavourable surgery that showed candidate clusters next to the surgery

The sample included in Step 1 consisted of 14 patients, with 10 patients having favourable outcome (see Tables 1 and 3). In patients with favourable outcome, 8 of 10 solutions were within the resection area (Fig. 2 and top of Table 3) when evaluating for performance (that is whether the candidate clusters were within the resection cavity). The remaining patients showed different profiles, with patient 14 (Fig. 3A) showing a candidate cluster at the anterior limit of the resection cavity, and patient 9 showing a candidate outside the resection area. In patients with unfavourable outcome, three of four patients had candidate clusters outside the corresponding resection regions. The last patient (10) showed a cluster inside the resected region while Engel 3 was the patient outcome.

**Table 3:** Individual performance of the model

ID	Sample	Spikes used	Cluster transition type	Size	Frequency bands	Threshold	Surgery outcome	In/out surgery	Model performance
1	Step 1	131	D->D	18	6,7,8,9,10,11,12	0.18	Favourable	in	Success
2	Step 1	108	D->D	19	7,8,9,10	0.10	Favourable	in	Success
3	Step 1	32	S->S	15	7,8,9,10	0.89	Unfavourable	out	Success
4	Step 1	66	D->D	16	6,7,8,9	0.26	Favourable	in	Success
5	Step 1	89	D->D	16	7,8,9,10,11	0.14	Favourable	in	Success
6	Step 1	66	S->S	22	3,4,5,6,7	0.28	Unfavourable	out	Success
7	Step 1	22	S->S	18	7,8,9,10,11	0.75	Favourable	in	Success
8	Step 1	24	D->D	22	6,7,8,9,10	0.67	Favourable	in	Success
9	Step 1	30	D->D	16	8,9,10,11	0.48	Favourable	out	Failure
10	Step 1	64	D->D	24	7,8,9,10,11	0.32	Unfavourable	in	Failure
11	Step 1	50	D->D	16	9,10,11,12	0.31	Favourable	in	Success
12	Step 1	25	D->D	20	8,9,10,11	0.56	Favourable	in	Success
13	Step 1	28	S->S	5	3,4,5,6,7	0.86	Unfavourable	out	Success
14	Step 1	23	D->D	10	5,6,7,8	0.72	Favourable	out	**Failure**
15	Step 2	37	D->D	19	1,2,3,4	0.57	Favourable	in	Success
16	Step 2	28	D->D	16	8,9,10,11	0.37	Unfavourable	out	Success
17	Step 2	23	D->D	15	6,7,8,9	0.54	Unfavourable	out	Success
18	Step 2	20	D->D	32	6,7,8,9,10	0.82	Unfavourable	out	**Success**
19	Step 2	24	D->D	16	8,9,10,11	0.70	Unfavourable	out	Success
20	Step 2	20	D->D	15	8,9,10,11,12	0.55	Unfavourable	in	Failure
21	Step 2	28	D->D	27	7,8,9,10,11	0.39	Favourable	out	**Failure**
22	Step 2	20	D->D	16	8,9,10,11	0.36	Favourable	in	Success
23	Step 2	21	S->S	19	6,7,8,9,10	0.90	Unfavourable	out	Success
24	Step 2	46	S->S	5	1,2,3,4	0.92	Unfavourable	out	Success

**Figure 4:**
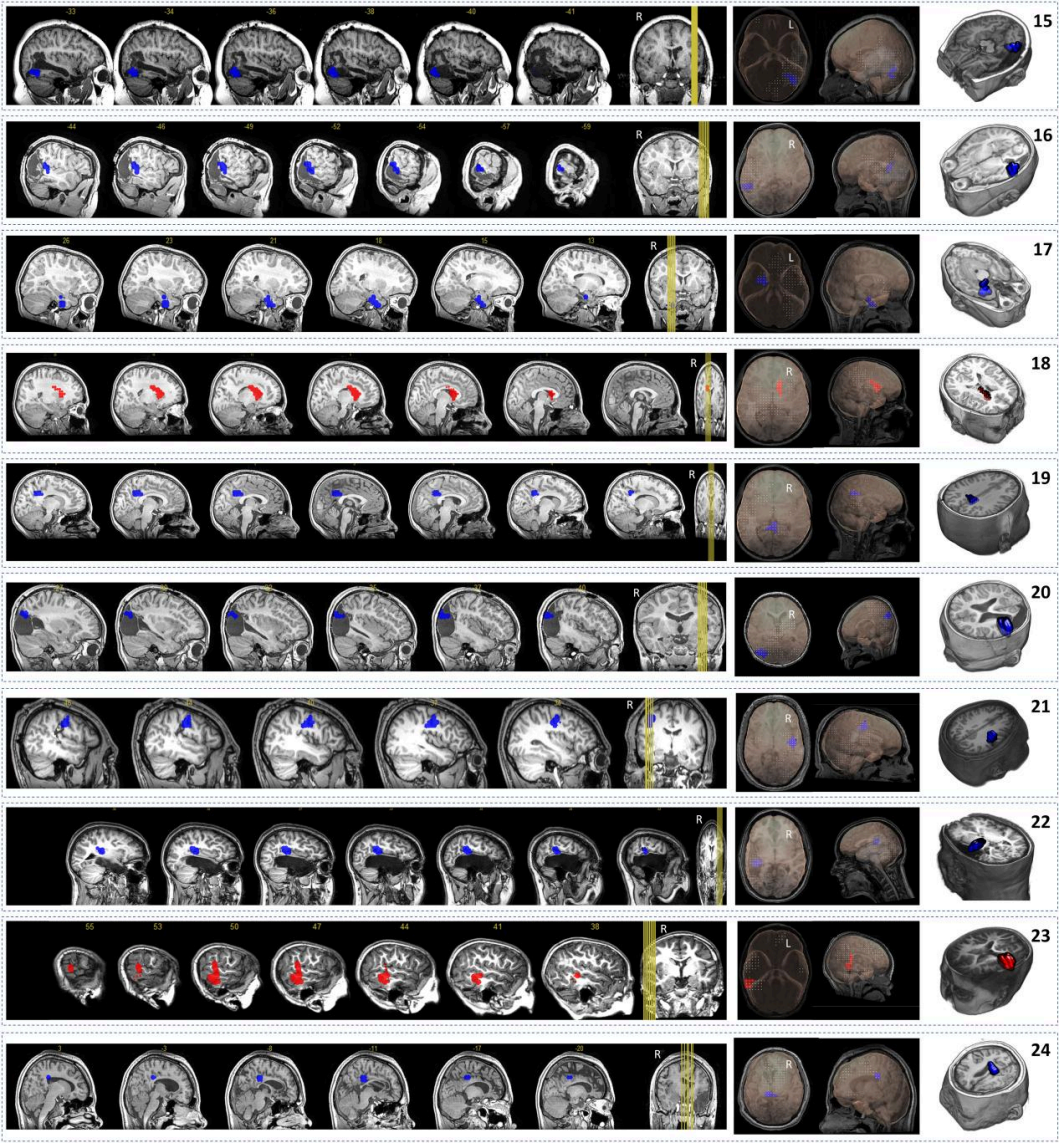
**Candidate clusters for the patients included in the Step 2.** The figure displays in each row the result for each one of the 10 patients involved in the second experiment (Step 2). All different views show the same information, the cluster location. In the centre figures, the 3D model shows the headmodel of the corresponding patient. Clusters in the rows 15, 16, 17, 19, 20, 21, 22 & 24 (highlighted in blue) indicate brain regions whose transition between the pre-spike period and the spike-onset period was defined by a desynchronizing–desynchronizing pattern. Clusters in the rows 18 & 23 (highlighted in red) represent brain regions with a synchronizing–synchronizing profile

Step 1 was used to develop the model. Step 1 was designed to emulate the testing of already performed surgeries. For patients included in Step 1, the network of interest included all nodes within the resected area, regardless of whether they were selected in the automatic network configuration. For Step 2 design, the network of interest was created by including only those nodes automatically selected. **Cluster transition type**: description of the transition profile (between pre-spike and spike) found in the nodes within the resulting cluster. **Size:** number of nodes of the cluster. When no clusters were found with a minimum size of 15 nodes, the process was repeated with 10 or 5 correspondingly. **Frequency bands:** the numbers indicate the bands where the cluster were found: (1) 4–8 Hz, (2) 8–14 Hz, (3) 15–30 Hz, (4) 30–45 Hz, (5) 45–55 Hz, (6) 65–75 Hz, (7) 75–85 Hz, (8) 85–95 Hz, (9) 95–105 Hz, (10) 105–115 Hz, (11) 125–135 Hz, (12) 135–145 Hz. **Threshold**: the value represents the minimum % of nodes that was needed to include to find the resulting cluster. **In/out surgery:** the column indicates whether the resulting cluster was located inside or outside the resection cavity. **Model performance**: success would indicate that the candidate cluster was found within the resection region in a seizure free patient after surgery or might be referring to those cases where the candidate cluster was found outside the resection region in a non-seizure free patient after surgery. Failure refers to a situation where the cluster was found outside the resection cavity in a patient with favourable outcome. If Success/Failure is highlighted with bold letters, the corresponding patient had a solution that was found in the border of the resected volume. These patients are displayed in Fig. 4.

D, desynchronizing; S, synchronizing; B, bulk.

In summary, the performance of the model reached the following values (see Table 4): sensitivity 80%, specificity 75% and accuracy 79%. In most cases (see Table 3), the frequency bands involved in the solutions ranged from 85 to 135 Hz, with two patients having frequency bands in the low medium range from 15 to 85 Hz (patients 6, 13). Candidate clusters remained stable for a minimum of 4-consecutive frequency bands in all patients by definition and reached a maximum of 7-consecutive frequency bands (Patient 1).

**Table 4:** Overall model performance

	True positives Fav + In	False negatives Fav + Out	True negatives unFav + Out	False positives unFav + In	Accuracy	Sensitivity	Specificity	Fisher test
Step 1 (14 patients)	8	2	3	1	0.79	0.80	0.75	0.0949
Step 2 (10 patients)	2	1	6	1	0.80	0.67	0.86	0.1833
All (24 patients)	10	3	9	2	0.79	0.77	0.82	0.0123

Fav, favourable outcome: Engel classification 1 after 2 years, from Durnford *et al.*^36^ UnFav, unfavourable outcome: Engel > 1. In/Out, model solution located inside/outside the resected cavity. The contingency tables used for the computation of the Fisher’s exact test for each sample were constructed using the data showed in the columns 2–5.

#### Virtual resection properties

Transition profiles of candidate clusters were analysed. In 10 patients with favourable outcome, the *desynchronizing*–*desynchronizing* transition profile was frequently observed and was found in 9 of 10 patients, whereas *synchronizing-synchronizing* transition profile was found in 1 patient (patient 7). In four patients with unfavourable outcome, *synchronizing*–*synchronizing* transition profile was found in three of four patients, and *desynchronizing*–*desynchronizing* transition profile was found in one patient (patient 10), for whom the model failed. No other transition profiles were found.

The thresholds used in the sequential process to rank the clusters (% of sources included in the model based on their corresponding CC score) were analysed. In patients with favourable outcome where a solution was within the resection, the average threshold was approximately 0.35. The average threshold was around 0.65 for patients with unfavourable outcome.

### Step 2

#### Blinded test of model performance

The sample included in Step 2 consisted of 10 patients, with 3 patients having favourable outcome (see Tables 2 and 3; Fig. 4). In patients with favourable outcome, two of three had solutions within the resection area. The remaining patient showed a candidate cluster that overlapped the border of the resection cavity (patient 21, Fig. 3B). In patients with unfavourable outcome, six of seven showed solutions that were located outside the resection area, with some patients having a cluster located in the periphery of the resection cavity (patient 18, Fig. 3C).

In summary, the performance of the model reached the following values (Table 4): sensitivity 67%, specificity 86% and accuracy 80%. The frequency bands behaviour mirrored Step 1, with most cases showing high frequency bands ranging from 65 to 145 Hz (Table 3, bottom), with two patients having comparatively lower frequency bands ranging from 4 to 45 Hz (patients 15, 24). Candidate clusters remained stable for a minimum of four consecutive frequency bands in all patients by definition and reached a maximum of five consecutive frequency bands.

#### Virtual resection properties

Transition profiles of candidate clusters were analysed. In three patients with favourable outcome, the desynchronizing-desynchronizing profile was found in all patients. In seven patients with unfavourable outcome, desynchronizing–desynchronizing profile was found in five patients, whereas synchronizing-synchronizing profile was found in two patients. Similar to Step 1, no other transition profiles were found. In patients with favourable outcome, the solution was found for an average threshold of 0.47. On the other hand, the patients with unfavourable outcome showed a first solution when an average threshold of 0.71 was applied.

In summary, the performance of the model, when considering the whole sample, reached the following values (Table 4): sensitivity 77%, specificity 82% and accuracy 79%.

## Discussion

A personalized computational neurosurgery model, using MEG data, was evaluated as a potential tool for guiding epilepsy surgery. This model offers a framework to test and plan surgery approaches trying to improve accuracy for a successful resection. Multidimensional data aggregate, comprising of spatial locations, frequential ranges and temporal transitions between brain states, was obtained using to model two different clinical scenarios: (i) testing an already performed surgery by including in the model the information of the resected cavity and (ii) a blind approach where the model was not informed by where was located the removed brain area.

The approach developed in this study represents many continual advancements of previously published studies. Our computational neurosurgery model is based on a personalized approach focusing on a single case diagnosis, in contrast to group approach.^29^ This individualized approach aims to represent a clinical scenario where a patient-specific model would be developed from pre-operative data. This contrasts with group data or theoretical models, where the design is largely experimental. We strongly view that clinically reliable and relevant representations are required for the introduction of this method to clinical practice. This study further expanded the usage of MEG assessment of functional networks during resting state^17,52^ to spike-related functional network analysis. This development allows for analysis of transition states and assess epilepsy-specific pathologic activity. In contrast to studies using invasive intracranial EEG recordings,^29^ this method allows for a non-invasive analysis. Additionally, our approach is also more practical, as it utilizes interictal activities which are more frequent and can be performed outpatiently, in contrast to those that relied on ictal data that typically would require inpatient monitoring (plus our model can be directly applied to ictal activity).^22,29^

We used MEG, as it is employed in,^17,52^ to assess interictal resting state networks, but we were focused in assessing spike-related networks. There are also studies, that used seizure modelling powered by iEEG data, assessing which regions should be targeted to surgery.^18,19^ Another approach to perform virtual resection is the methodology applied in a large multi-centre study of the virtual brain platform.^21,53,54^ This methodology is based on the structural network enriched with multimodal clinical and functional information. In a very elegant study, An *et al*.^54^ have employed a modularity analysis to identify target brain regions and fibre tracts involved in seizure propagation, using The Virtual Brain platform. They validated the model via electrical stimulation for pre- and post-surgical condition to quantify the impact on the signal transmission properties of the network. In comparison, our study focused in giving useful information in the surgery planning using the minimal information and computational resources.

**Figure 5:**
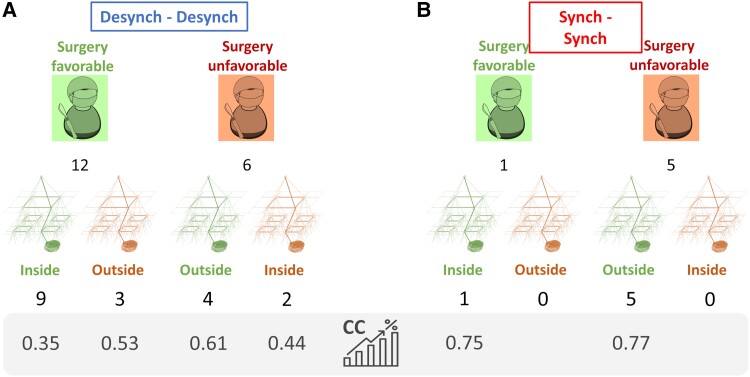
**Results overview.** (**A**) Results for the 18 patients whose first cluster described a *desynchronizing–desynchronizing* solution. Surgeon images depict the number of surgeries with each outcome. The pyramidal graphs show the location of the clusters with respect to the resection. Best model performance was obtained for those patients whose model reached to a solution with low threshold (that is the candidate clusters involved sources with high CC values). (**B**) Results for the six patients whose first cluster described a *synchronizing–synchronizing* solution. The model agreed in all cases, showing solutions with high thresholds that is the model needed to include more brain volume, by involving sources with lower CC values). The best prognosis would be given to those patients whose model solutions showed low thresholds and *desynchronizing–desynchronizing* transition profile

We appreciate the concepts of previous works,^29^ which suggested the role of distributed cortical network in seizure generation, and the effect of synchronizing and desynchronizing brain regions in seizure spread.^28,55^ Extending this previous knowledge, our study identified three relevant characteristics of candidate clusters: (i) frequency bands, (ii) transitional profile and (iii) solution threshold. The combination that most frequently emerged in successful models was as follows: high frequency bands, desynchronizing-desynchronizing transitional profile and lower solution threshold (see Fig. 5). These parameters were found in 13 of 24 patients and can be a useful feature to guide resection or placement of intracranial recording. Only six patients showed a synchronizing-synchronizing transition profile. In five cases (of 6), the surgical intervention was unfavourable, indicating that this transition profile would be associated with atypical cases, for whom, the surgery would be less effective.

This approach focusing on the core of robust dynamic behaviour using spatial and frequential homogeneity can potentially decrease the appreciation of perilesional synchronizing sources, as was described in Kini *et al*.^29^ However, we view that the clusters areas obtained through our method may be more easily applicable to epilepsy surgery, as our results provide a more clearly defined region of interest, which was created from inter-ictal activity obtained through non-invasive measure. Additionally, although smaller in size, the cluster candidates obtained through our approach appear clinically relevant, as most patients with favourable clinical outcome have candidate clusters localized within the resection cavity. Similarly, most patients with unfavourable outcome have clusters localized outside of the resection cavity. Two patients who received re-operation that included these clusters led to favourable outcome (patients 3–4 and 6–7). In contrast, two patients who received re-operation that did not include these clusters would continue to have unfavourable outcome (patients 16–17 and 18–19). These cases further reinforced the credibility of our model. However, despite our promising result, we view that an additional prospective study with a larger population is required to validate our approach.

In the study of Kini *et al.*, it is hypothesized that removing synchronizing nodes and sparing desynchronizing ones would be better for patients’ outcome. In our study, the patients whose solutions were desynchronizing - desynchronizing had in general better prognosis. Both studies have enough differences that could be explaining this apparent discrepancy. Kini *et al*. assessed, using group data, the profiles of the nodes found in the edges of the cavity, finding some desynchronizing nodes as well in the beta band. We can only hypothesize about why *desynch*–*desynch* nodes would be important for the spike generation. As pointed by,^29^ these nodes could be acting as controllers that modulate the activity of the surrounding area. Since this area was already removed, it is reasonable to think that the region would be playing a role on the malfunctioning of the electrophysiological activity. In a previous study,^22^ the authors reported that the seizure onset zone was composed by an insulated region that became more connected with the epileptic network with the development of the seizure. In our study, the candidate cluster always conformed a specific closed region, surrounded by other nodes of the network that did not behave the same way; our region appeared to be functionally insulated. This idea of a specific region, the epileptic onset zone, could be reflecting a controller tole type or as a hub of the network. Computational models have already evaluated the role of the hubs in virtual surgeries,^17,56-58^ and the results stated that removing hubs of the epileptic network would be an optimal strategy that reach a successful outcome. This fact could be driven by the well-known disrupted GABA transmission in epilepsy. The existence of regions with an epileptic-related stable dynamic, characterized by desynchronization in both brain states, could be indeed affected by genetic factors,^59^ which seems to downregulate the normal GABA signalling.^60^

The predominance of high frequency bands of candidate clusters in this study is consistent with prior literature.^61^ There is mounting evidence regarding the relevance of characterizing epileptogenic zones through high frequency oscillations.^9,62^ Velmurugan *et al*. reported that the presence of MEG high frequency activity associated with the resected cortex showed a high accuracy (78.8%) in predicting a patient's outcome. Furthermore, the overlapping of the high frequency sources with the resected brain region is associated with successful surgical outcome (82.4%) when compared to non-overlap group (14.3%). In this study, we also identified high frequency activity in the majority of candidate clusters with accurate outcome (Step 1: 85%; Step 2: 80%). This result further added to evidence towards relevance of high frequency activity in electrophysiological recordings for epilepsy surgery.

This MEG-derived model can provide both personalized candidate clusters and functional assessment of transitional network behaviour. Additional benefit of MEG in functional network analysis is its ability to provide greater coverage of cortical networks compared to invasive intracranial recordings. Furthermore, although this protocol was designed for MEG recordings, we believe that could be as well applied to electroencephalographic data upon improvement of volume conductor models^63^ and recording spatial resolution.^64^ Currently, the application of epileptic network analysis through MEG can be viewed as complementary to SEEG. From the evidence that combined MEG-SEEG data could offer optimal results in capturing epileptic activity,^65^ we also perceive that there is potential for integrated MEG-SEEG data in the study of epileptic network connectivity.

MEG has some limitations as an acquisition method, as sources with adequate signal-to-noise are required for successful modelling, and models created from low signal-to-noise sources would not provide accurate results. Epileptic activity that has widespread distribution would also lack reliable solutions under the model since it is designed to work with the time evolution of the spike-related activity. Notwithstanding, in pure generalized epilepsies, the removal of the nodes spreading the epileptogenic activity, could be helping on stopping the diffusion of the epileptogenic activity and consequently helping on seizures relief.^54^ Another limitation is the lack of simultaneously recorded EEG leads to an underestimation of epileptiform activity because unique EEG discharges are not captured. Co-registration of MEG dipoles to MRI must also take typical spatial alignment error into account, where up to 5 mm deviation can be expected. Another important factor is the interpretation of the solutions in the successful patients. In all cases, the solutions were significantly smaller than the resection cavity. We cannot prove whether it would have been enough to resect only those portions instead the whole cavity. In a similar way, we cannot ensure the results for the unsuccessful patients since the proposed solutions were not in the resected areas. It would be speculative to interpret that the localization represents the actual region where surgery should have been performed. A retrospective study it is just a validation. A class-I prospective study should be the only way to have a real value of the model in the clinical scenario.

Considering these limitations, the three instances where our model fails require specific clinical discussion (Step 1: patients 9, 10; Step 2: patient 20.). Clear model failure exists in patient 9 who achieved a favourable outcome after resection of focal cortical dysplasia further away from the candidate cluster. Patients 10 and 20 had unfavourable outcome despite candidate clusters within the resection cavity. In cases 10 and 20, the clusters of the model were located within the resection cavity, but the surgery outcome was unfavourable. Both of these patients had initial improvements but later developed increased seizures, which may indicate progressive reorganization of the epileptogenic network that could not be easily predicted by a computational model.

Two cases were difficult to classify as a model success or failure (patients 14, 21) due to the clusters being localized to the immediate vicinity of resection margin. These cases could debatably be considered model failures as the patients were seizure free despite the clusters being adjacent to resection. However, we view that two potential hypotheses exist that can support the possibility of model success. These are scenarios where the surgery either (i) creates functional disconnection between the clusters and surrounding cortex or (ii) causes destruction of the peri-resectional cortex where the clusters are localized. We also must take typical spatial alignment error into account, as previously described in limitations of MEG.

## Conclusion

This is the first personalized data-driven computational neurosurgery model for epilepsy surgery using MEG data. The method was validated against the epilepsy surgery outcome. The candidate clusters with high frequency bands, stable transitional profile, and lower solution threshold are associated with favourable post-operative outcome. Although the model was tested in a limited number of epileptic patients, it provides a reliable result even in a cohort containing variable epilepsy localization and syndromes. This model should be prospectively studied, where the method can be further optimized, its clinical use further validated. We view that the utilization of this model has significant potential in improving the outcome of epilepsy surgery.

## Data Availability

The data and code that support the findings of this study are available from the corresponding author, upon reasonable request. All the algorithms used in the present paper are available at https://osf.io/e2xrt/.

## Abbreviations

CC =: control centrality
FC =: functional connectivity
MEG =: magnetoencephalography
PLV =: phase locking value
S =: synchronizability
SAN =: spike-associated network
SEEG =: stereoelectroencephalography
VCR =: virtual cortical resection
